# Genome-wide identification, comparative analysis and functional roles in flavonoid biosynthesis of cytochrome *P450* superfamily in pear (*Pyrus* spp.)

**DOI:** 10.1186/s12863-023-01159-w

**Published:** 2023-10-03

**Authors:** Wei Zhang, Hongxiang Li, Qionghou Li, Zewen Wang, Weiwei Zeng, Hao Yin, Kaijie Qi, Ying Zou, Jian Hu, Baisha Huang, Peng Gu, Xin Qiao, Shaoling Zhang

**Affiliations:** Sanya Institute of Nanjing Agricultural University, State Key Laboratory of Crop Genetics & Germplasm Enhancement and Utilization, College of Horticulture, Nanjing Agricultural University, Nanjing, 210095 China

**Keywords:** Pear, Cytochrome *P450*, Comparative analysis, Functional roles, Flavonoid biosynthesis

## Abstract

**Background:**

The cytochrome *P450* (CYP) superfamily is the largest enzyme metabolism family in plants identified to date, and it is involved in many biological processes, including secondary metabolite biosynthesis, hormone metabolism and stress resistance. However, the *P450* gene superfamily has not been well studied in pear (*Pyrus* spp.).

**Results:**

Here, the comprehensive identification and a comparative analysis of *P450* superfamily members were conducted in cultivated and wild pear genomes. In total, 338, 299 and 419 *P450* genes were identified in Chinese white pear, European pear and the wild pear, respectively. Based on the phylogenetic analyses, pear *P450* genes were divided into ten clans, comprising 48 families. The motif and gene structure analyses further supported this classification. The expansion of the pear *P450* gene family was attributed to whole-genome and single-gene duplication events. Several *P450* gene clusters were detected, which have resulted from tandem and proximal duplications. Purifying selection was the major force imposed on the long-term evolution of *P450* genes. Gene dosage balance, subfunctionalization and neofunctionalization jointly drove the retention and functional diversification of *P450* gene pairs. Based on the association analysis between transcriptome expression profiles and flavonoid content during fruit development, three candidate genes were identified as being closely associated with the flavonoid biosynthesis, and the expression of one gene was further verified using qRT-PCR and its function was validated through transient transformation in pear fruit.

**Conclusions:**

The study results provide insights into the evolution and biological functions of *P450* genes in pear.

**Supplementary Information:**

The online version contains supplementary material available at 10.1186/s12863-023-01159-w.

## Background

Cytochrome *P450* monooxygenase (CYP), a type of heme-thiolate protein, belongs to a multigene superfamily with an ancient origin. And it was named *P450* because its carbon-monoxide-binding form has a signature absorption peak at 450 nm [1]. As one of the largest enzyme protein superfamilies, *P450* family genes have been discovered in a variety of organisms, which include plants, animals, fungi, protists, archaea, bacteria and even viruses [2]. *P450*s use NADPH and oxygen to catalyze various reactions such as sulfoxidation, dealkylation, dehalogenation, epoxidation, ring extension and reduction [3, 4]. In addition, most plant *P450*s are membrane-bound enzymes anchored in the endoplasmic reticulum membrane through a N-terminal hydrophobic signal sequence [5]. In plants, *P450* genes are involved in the biosynthetic processes of some biological molecules, such as flavonoids, isoflavonoids, sterols, phytoalexins, fatty acids, plant hormones, signaling molecules and structural polymers, like lignins [4]. *P450* genes play significant roles in plant growth and development and improve plant resistance to various biotic and abiotic stresses.

Strict standards for *P450* gene identification and classification have been set [6, 7]. A protein is considered a P450 protein if it contains the following four conserved regions: heme‐binding motif, K‐helix region, PERF region and I‐helix motif [8]. The *P450* proteins can be divided into A-type and non-A-type clades according to the diverse representative motif signatures. For the heme-binding region, A-type CYPs are “PFGXGRRXCXG”, whereas non-A-type CYPs are “XFXXGXRXCXG”. The sequences of the PERF motifs of A-type and non-A-type CYPs are “FXPERF” and “FXPXRX”. The I-helix motifs of A-type CYPs are “AGXDT”, whereas they are “AGX[D/E]T” in non-A-type CYPs [9]. The classification criteria for *P450* genes are mainly based on phylogenetic and homologous sequence analyses [6]. According to the phylogenetic trees, deep clades are represented by different clans. At present, 11 *P450* gene clans, including 7 single-family clans (CYP51, CYP74, CYP97, CYP710, CYP711, CYP727 and CYP746), 4 multiple-family clans (CYP71, CYP72, CYP85 and CYP86) have been defined in plants [10]. In general, A-type correspond to the CYP71 clan, non-A-type correspond to all other clans. Furthermore, if two *P450* sequences share more than a 40% amino acid sequence similarity, then they are recognized as being in the same *P450* family. If the sequence similarity is greater than 55%, then they are considered to be in the same subfamily. If the sequence similarity exceeds 95%, then they are allelic variants. However, if they share a 40% or less identity, then they may represent new *P450* families [11, 12]. So far, 670 *P450* families have been named in plant kingdom [5]. Among them, 52 families have been widely detected in angiosperms and recognized as core *P450* families [13]. The discovery of a new *P450* family has been rarely reported in recent years.

Owing to the sequencing of various plant species’ genomes, more *P450* genes have been identified on a genome level. The presence of a plant *P450* gene was firstly reported in cotton in 1969 [14]. In 1990, the *P450* gene sequence associated with avocado ripening, CYP71A1, was cloned [15]. In recent years, the *P450* gene family has been identified in many plants, such as Arabidopsis (*Arabidopsis thaliana*), rice (*Oryza sativa*), sacred lotus (*Nelumbo nucifera*), papaya (*Carica papaya*), mulberry (*Morus notabilis*) and grape (*Vitis vinifera*) [7, 11, 13, 16, 17]. By 2018, an estimated 16,000 P450 sequences had been annotated and named in plants [18]. The number of *P450* genes identified in recent years has increased sharply. Plant *P450* superfamily members are estimated to account for approximately 1% of genes per whole-genome in a given plant species [8, 13].

Different *P450* families or subfamilies have distinct functions and play different roles in secondary metabolite synthesis, plant growth and development and stress resistance. For instance, several members of the CYP78A subfamily regulate the size of Tartary buckwheat fruit [19]. The CYP71 family members in pepper protect the fruit from pathogen infection [20]. The CYP719 and CYP80 family genes in sacred lotus are involved in the synthesis of benzylisoquinoline and aporphine alkaloids [16]. The CYP93C subfamily in *Medicago sativa* is related to isoflavone biosynthesis [21]. The CYP75 family participates in anthocyanin synthesis processes in *Hordeum vulgare*, resulting in the synthesis of different anthocyanin compounds in different plant parts [22]. However, there are also some members of the same family being involved in different pathway. The CYP51 family has a pivotal role in triterpenoid biosynthesis in tobacco [23], whereas a member of this family can also encode a obtusifoliol 14α-demethylase enzyme to involve in the sterol-specific pathway [24]. The CYP81D subfamily genes may confer salinity tolerance to bread wheat [25], whereas the CYP81F family involved in the biosynthesis of 4-hydroxy-3-indolylmethyl glucosinolate and 4-methoxy-3-indolylmethyl glucosinolate [26].

Flavonoids are an important class of polyphenol secondary metabolites in plants that have strong biological activities. They play crucial roles that not only affect fruit color and flavor, but also increase plant resistance to stress, disease and insect pests [27, 28]. Moreover, they are the main biochemical and pharmacological components in many edible plants and have anti-cancer, hypoglycemic, anti-viral, anti-inflammatory characteristics. They are used in the treatment of cardiovascular diseases and Alzheimer’s disease, and they have other biological activities beneficial to the human body [29–32]. With the increased interest in healthy diets, consumers pay more attention to the nutrition and health-care functions of fruits, in addition to the appearance and flavor. Flavonoids are important components of fruit nutrition and health functions, and they play very vital roles in fruit nutritional quality. *P450* genes participate in the biosynthesis of flavonoids. The members of the CYP75 family encode enzymes, including flavonoid 3’-hydroxylase (F3’H) and flavonoid 3’,5’-hydroxylase, that play crucial roles in the flavonoid synthetic pathway [33]. The CYP75A subfamily is necessary for the formation of tricin because it is capable of catalyzing the conversion of apigenin [34]. The CYP93 family contains many key enzymes, including flavone synthase II, (2S)-flavanone 2-hydroxylase and 2-hydroxyisoflavanone synthase, which are involved in flavone and isoflavone biosynthesis [35]. In soybean (*Glycine max* L.), CYP93B16 is a flavone synthase II that can convert flavanones into flavones [36]. According to a previous study, the CYP93C subfamily genes participate in the construction of the isoflavonoid skeleton by catalyzing the hydroxylation of the flavanone molecule at C-2 and the intramolecular 1,2-aryl migration from C-2 to C-3 [37]. In sweet basil (*Ocimum basilicum* L.), CYP82D catalyzes the 6-hydroxylation of a 7-O-methylated precursor, which is an important reaction in flavone biosynthesis [38].

Pear is a crucial economic fruit tree, and it is also a temperate Rosaceae fruit species. It has a long cultivation history, with the earliest occurrence dating back to more than 30,000 years ago [39]. The *Pyrus* genus contains a variety of pear species, including *Pyrus bretschneideri* (Chinese white pear), *Pyrus communis* (European pear), *Pyrus ussuriensis*, *Pyrus pyrifolia* and *Pyrus sinkiangensis*. Chinese white pear (‘Dangshansuli’), European pear (‘Bartlett’) and a wild pear species (Pyrus betuleafolia, ‘Shanxi Duli’) are very representative pear species and their genomes have been published. [39–41]. The sequencing of pear genomes provides a foundation for pear genomics and molecular biology studies. In this research, we identified the whole-set of *P450* superfamily genes in these pear genomes, and we resolved the evolutionary expansion and expression patterns. We then screened for the *P450* members involved in flavonoid biosynthesis. The preliminary functional role of a candidate gene was revealed using transcriptome, qRT-PCR and transient transformation analyses. This study provides a new perspective on the evolution and biological functions of pear *P450* genes.

## Results

### Identification of *P450* genes in pear (*Pyrus* spp.)

**Fig. 1:**
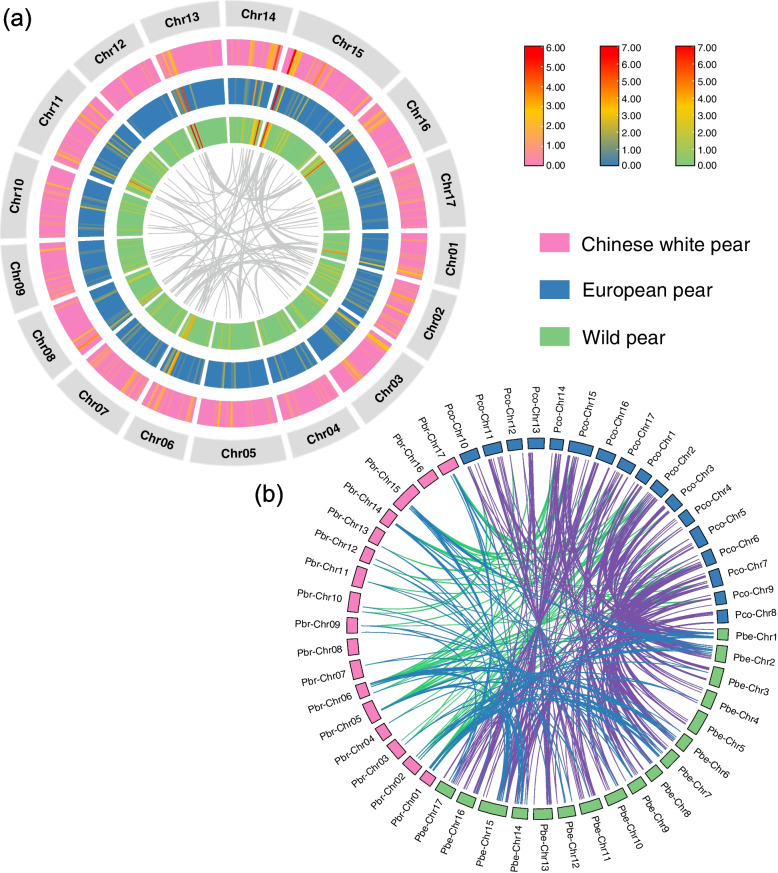
Gene localization and syntenic relationships of *P450* genes among three pear species. **a** The chromosomal distribution of *P450* genes in three pear species. The circle diagram from outside to inside is chromosome, density of *P450* genes in Chinese white pear, European pear and wild pear respectively. The innermost links are the syntenic pairs of *P450* genes in Chinese white pear. **b** The syntenic relationships of *P450* genes among three pear species. The chromosomes of three pear species are distinguished by different colors. Purple links represent syntenic pairs between European pear and wild pear, cyan links represent syntenic pairs between European and Chinese white pear, and blue links represent syntenic pairs between Chinese white pear and wild pear

338, 299 and 419 *P450* genes were identified in Chinese white pear (named *PbCYP*s), European pear and wild pear respectively. The basic physical and chemical properties of the *PbCYP* genes, including gene length, amino acid length, protein MW (molecular weight), pI (isoelectric point) and gene location, were analyzed (Supplementary Table 1a—c). Both the length of amino acids, MWs and pIs of *P450* genes in pear showed great variations with over tenfold changes between the shortest and longest sequences. The lengths of majority of *P450* proteins were about 500 amino acids (Supplementary Fig. 1). In Chinese white pear, amino acids length ranged from 104 amino acids (Pbr012516-v2.1, named PbCYP716C7) to 1,258 amino acids (Pbr028150-v2.1, named PbCYP749A6), and their MWs ranged from 11.54 kDa to 143.69 kDa. In European pear, amino acid length ranged from 103 (pycom02g24890) to 1505 (pycom17g18000), and their MWs ranged from 11.39 kDa to 174.29 kDa. In wild pear, amino acid ranged from 101 (Chr14.g50060) to 1352 (Chr15.g04620), and their MWs ranged from 11.54 kDa to 152.24 kDa. The ranges of pIs of *P450* proteins in three pear species are located in 4.41–11.2, 4.47–10.6 and 4.29–11.56 respectively. The distribution of the *P450* genes along the 17 pear chromosomes was uneven. The chromosomal distribution of *P450* genes across three pear species exhibited similar pattern (Fig. 1a and Supplementary Fig. 2). Chromosome (Chr) 15 contained the highest number of *P450* genes (37–56 genes), while Chr12 contained the lowest number of *P450* genes (2–8 genes) in three pear genomes (Supplementary Table 2). In Chinese white pear, 24 gene clusters were found in 12 out of 17 chromosomes, and the number of genes within these clusters ranged from three to nine (Supplementary Table 3 and Supplementary Fig. 2). In European pear and wild pear, 30 and 38 gene clusters were found respectively. A high density of *P450* genes was found in the end region of Chr14 and the start region of Chr15. 6, 6 and 4 gene clusters were observed in Chr15 of three pear species respectively, and they mainly resulted from tandem duplication (TD) or proximal duplication (PD). In addition, four clusters containing 20 genes were found in the first 12 Mb of Chr13 in European pear.

### Phylogenetic analysis of *P450* genes

**Fig. 2:**
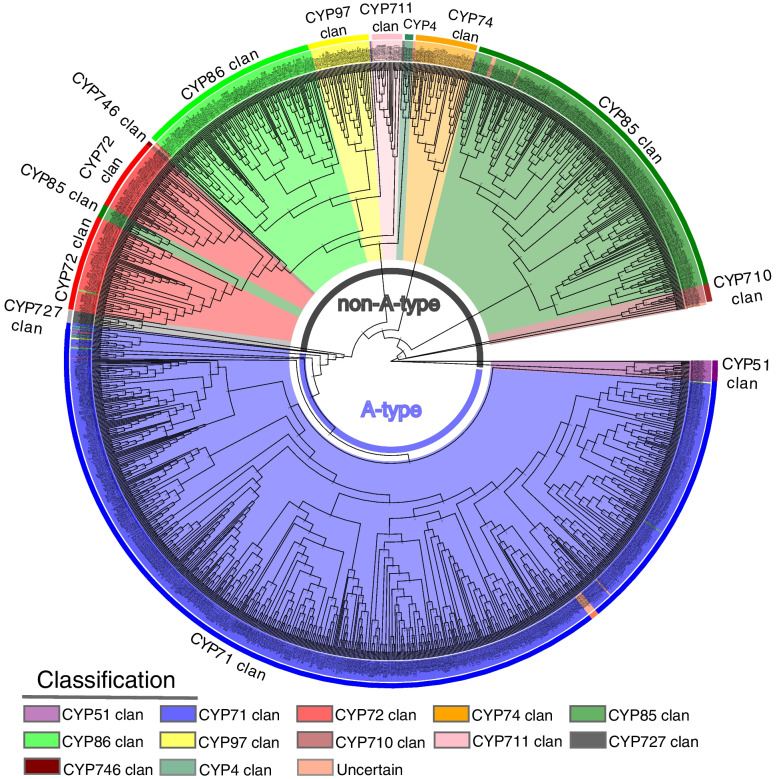
Phylogenetic analysis of Chinese white pear P450 sequences and representative protein sequences of each P450 subfamilies from different species. A maximum-likelihood phylogenetic tree was constructed using IQ-TREE with 1000 bootstrap replicates. Different clans of *P450* genes are indicated with different colors: seagreen, CYP4 clan; purple, CYP51 clan; blue, CYP71 clan; red, CYP72 clan; orange, CYP74 clan; green, CYP85 clan; lime, CYP86 clan; yellow, CYP97 clan; brown, CYP710 clan; pink, CYP711 clan; turq, CYP746 clan; coral, uncertain. Above protein sequences can be obtained from Figshare website (https://doi.org/10.6084/m9.figshare.19595299.v1)

According to the 1105 *P450* protein sequences of different species and Chinese white pear, a phylogenetic tree was constructed using the maximum-likelihood (ML) method (Fig. 2). Another *P450*s phylogenetic tree of Chinese white pear and four Rosaceae species (*Malus domestica*, *Fragaria vesca*, *Prunus persica* and *Prunus mume*) was constructed to further modify the classification of pear *P450* genes (Supplementary Fig. 3). Finally, Chinese white pear cytochrome *P450* genes were divided into two major clades: A-type (45.0%, 152/338) and non-A-type (55.0%, 186/338) in accordance with the classification of *P450* genes in different species (Supplementary Table 4a—f). The two clades were further grouped into ten clans (CYP51, CYP71, CYP72, CYP74, CYP85, CYP86, CYP97, CYP710, CYP711 and CYP727), comprising 48 families. Among them, the CYP71 clan, which contains 19 families (CYP71, CYP73, CYP75–79, CYP81–82, CYP84, CYP89, CYP92–93, CYP98, CYP701, CYP703, CYP706, CYP712 and CYP736), is the largest clan, making up the whole A-type clade, and the remaining nine clans, which include 29 families (CYP51, CYP72, CYP74, CYP85–88, CYP90, CYP94, CYP97, CYP704, CYP707, CYP710, CYP711, CYP714–716, CYP718, CYP720–722, CYP724, CYP727–729, CYP733–735 and CYP749) belong to the non-A-type clade. Four CYP clans (CYP71, CYP72, CYP85 and CYP86) contain multiple families, and six CYP clans (CYP51, CYP74, CYP97, CYP710, CYP711 and CYP727) each contain a single family.

The *P450* protein sequences of European pear and the wild pear were compared with those of Chinese white pear by BLASTP respectively. Similarly, the *P450* genes in the European pear and wild pear were also divided into ten clans (Supplementary Table 4c—f). A phylogenetic tree was also constructed using *P450* protein sequences from Chinese white pear, European pear and wild pear, in which *P450* genes classified into the same clan among three pear species were well clustered, supporting the classification results based on BLASTP alignment (Supplementary Fig. 4). The European pear genome only contains 42 out of 48 CYP families identified in the Chinese white pear and wild pear. It is lacking the CYP87, CYP703, CYP718, CYP724, CYP733, and CYP735 families. In total, 51.8% of *P450* genes in European pear (155/299) and 53.7% of *P450* genes in the wild pear (225/419) belong to the A-type genes (CYP71 clan), which is higher than in Chinese white pear (45.0%). The CYP71 family has the largest number of genes in Chinese white pear, European pear and the wild pear. Seven families (CYP75, CYP703, CYP710, CYP712, CYP720, CYP727 and CYP733) have only one member in Chinese white pear. Similarly, eight (CYP51, CYP710, CYP712, CYP720–CYP722, CYP727 and CYP729) and seven (CYP87, CYP703, CYP710, CYP712, CYP720, CYP727 and CYP733) families only contain one gene in European pear and the wild pear, respectively (Supplementary Table 5).

We also collected *P450* gene members belonging to different clans and families from eight plant species, *A. thaliana* (272), *Citrus clementina* (296), *M. notabilis* (174), *V. vinifera* (316), *C. papaya* (142), *S. lycopersicum* (457), *O. sativa* (334) and *N. nucifera* (172), and compared them with *Pyrus* species [7, 11, 13, 16, 42, 43] (Supplementary Table 5)*.* The number of A-type (CYP71 clan) *P450* genes in most of the 11 species accounted for more than 50% of all *P450* genes, except for Chinese white pear, papaya and lotus in which 45.0%, 49.3% and 37.2% belonged to the A-type genes, respectively. The number of genes in the same CYP family varied greatly among different plant lineages. The CYP51 (10 genes) and CYP709 (9 genes) families showed expansion in the monocot species rice, compared with 10 other eudicot species that contained less than 4 genes. In particular, the CYP99 and CYP723 families were only found in rice, indicating that they may be monocot-specific CYP families. In sacred lotus (*N. nucifera*), a basal eudicot, the CYP80, CYP82 and CYP716 families have expanded compared with in rice. The CYP80 family may be involved in the biosynthesis of alkaloids in lotus [16]. CYP82 family members are linked to homoterpene and flavonoid metabolisms, which are associated with defending against biotic and abiotic stresses [44]. The roles of CYP716 family members in gibberellin biosynthesis have been verified in early-diverging plants (moss and selaginella), gymnosperms and angiosperms [45]. The CYP702, CYP705 and CYP708 families have only been detected in Arabidopsis, indicating that they may be Brassicales-specific CYP families [5, 10]. The CYP702 family may be associated with triterpenoid metabolism, and this may be related to production of special metabolites in Brassicale species [10]. The maximum number of known *P450* genes exists in tomato, perhaps due to its large genome size (900 Mb) and high percentage of the pseudogenes [43].

### Analyses of *P450* conserved motifs and gene structures

**Fig. 3:**
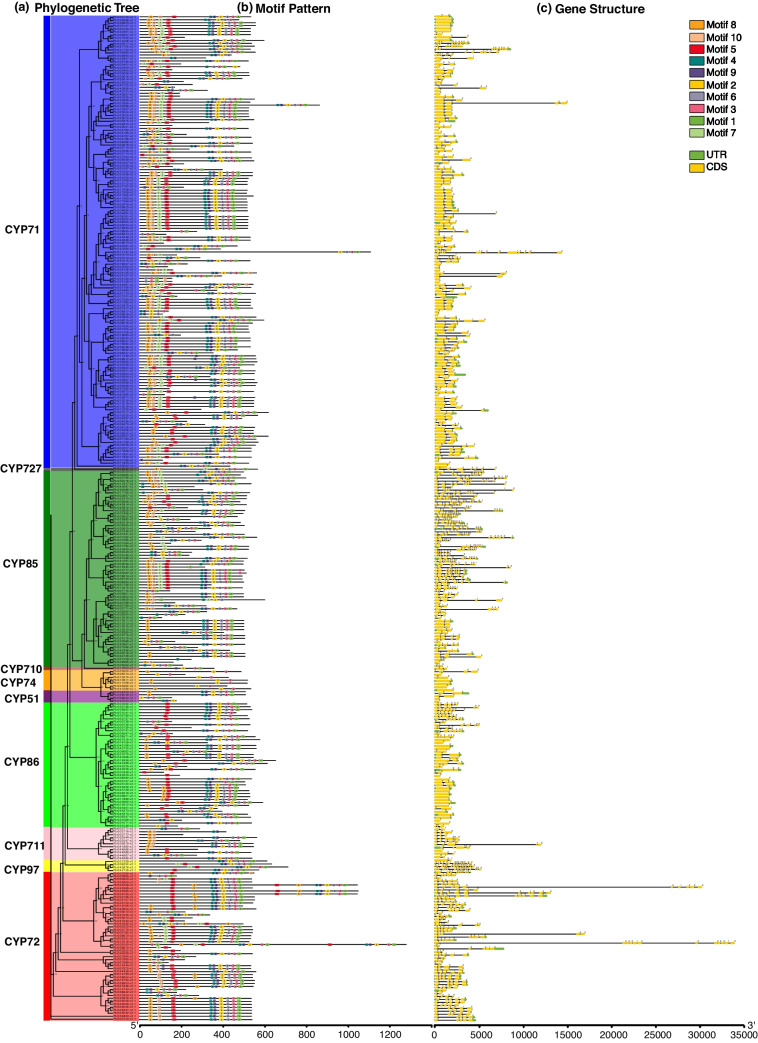
Phylogeny, conserved motifs and gene structures of *P450* genes in Chinese white pear. **a** The phylogenetic tree of the full-length sequences of Chinese white pear P450 proteins was constructed using IQ-TREE. **b** Motif compositions of the P450 proteins in Chinese white pear. MEME tools were used to identify motifs. Ten motifs (motifs 1–10) are indicated by different colors. The scale below represents the length of a protein. **c** Gene structures of *P450* genes in Chinese white pear. The green boxes indicate untranslated 5′- and 3′-regions, and the black lines indicate introns. The numbers indicate the phase of the corresponding intron

To further resolve the characteristics of pear *P450* genes, conserved motifs and exon–intron structures were investigated. We used the MEME online tool to detect conserved motifs of the *P450* family genes in Chinese white pear (Fig. 3 and Supplementary Table 6b), European pear (Supplementary Fig. 5 and Supplementary Table 6c), wild pear (Supplementary Fig. 6 and Supplementary Table 6d) and all three pear species (Supplementary Fig. 7 and Supplementary Table 6a). Ten conserved motifs were identified in three pear species respectively. The motif with similar sequence characteristics across three pear species can be observed although motif number is not always same such as motif 1, motif 2 and motif 1 in Chinses white pear, wild pear and European pear respectively. Here, *P450*s in Chinese white pear were used as a representative to clarify the results from conserved motifs and gene structures.

Similar distribution patterns of conserved motifs were found in members within the same clan or family (Fig. 3a and b). Motifs 1–4 correspond to four signature regions in the amino acid sequences, the heme‐binding region, K‐helix motif, PERF region and I‐helix motif, and these are the typical features of *P450* family genes (Supplementary Table 6). Motifs 1 and 3 are located at the C-terminal regions of CYPs, and motifs 2 and 4 are close to motifs 1 and 3, respectively. Approximately 91.1% (308 of 338) of the *P450* genes contained at least one of these four motifs. Motifs 6 and 9 were also found in most genes, at 78.1% (264 of 338) and 81.1% (274 of 338), respectively. Approximately 52.6% (80 of 152) of CYP71 clan members contained all 10 conserved motifs. The numbers of motifs in the CYP710 and CYP74 clans were fewer, having only 2–4 motifs. Majority of families in the CYP85 clan did not have motif 5 or motif 7 except for CYP707 family. All the CYP51 clan members lacked motifs 5, 7 and 10. The majority of families (CYP86, CYP94 and CYP704) in the CYP86 clan did not have motifs 7 and 10. Similarly, the absence of motif 7 or motif 10 was observed in some families (CYP714, CYP715, CYP734 and CYP735) of the CYP72 clan. Motif 5 or 7 was absent in most of the CYP711 clan members. And 84% (42 of 50) of the CYP72 clan members contain motif 5. Thus, different CYP clans had distinct conserved motif patterns, but those in the same clan presented similar features. This supported classification results from the phylogenetic analysis.

To further explore the gene features of *P450* family members in Chinese white pear, their exon–intron structures were analyzed (Fig. 3c). The number of introns in the *PbCYP*s varied from 0 to 15. A high proportion of genes (94/152) in the CYP71 clan was found to have one intron. The CYP74 and CYP86 clans had higher percentages of single exon genes (71.4% and 42.9%, respectively). CYP85, CYP97 and CYP72 clan members presented more complex gene structures, incorporating multiple introns and exons (54/66, 4/4 and 42/50, respectively). The majority of CYP85 clan members had 1 to 9 introns. CYP97 clan members contained 9 to 15 introns, and CYP72 clan members contained 1 to 11 introns.

### Collinearity and gene duplication event analyses of *P450* family genes

**Fig. 4:**
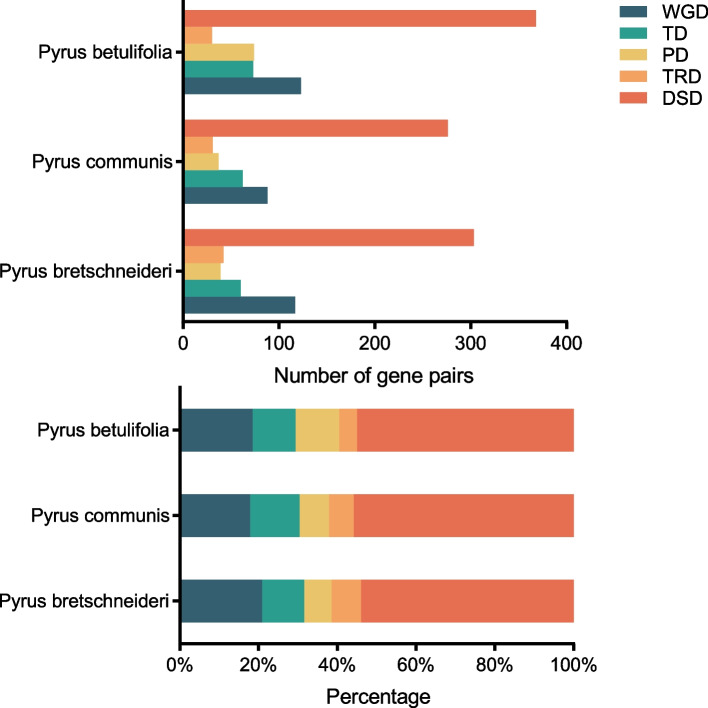
Comparison of *P450* gene pairs derived from five modes of gene duplication in *Pyrus bretschneideri*, *P. communis* and *P. betulifolia.* The *x*-axis in the upper histogram represents the numbers of *P450* gene pairs. The *x*-axis in the lower histogram represents the percentage of each duplication events. The *y*-axes of both histograms show the three pear species. Different duplication events are represented by different color bars. Whole genome duplication (WGD), tandem duplication (TD), proximal duplication (PD), transposed duplication (TRD), dispersed duplication (DSD)

Gene duplication is a significant booster of gene family expansion and gene functional diversification, and it can occur through different mechanisms, including whole-genome (WGD), TD, PD, transposed (TRD) and dispersed (DSD) duplication [46]. To unravel the evolutionary history of the *P450* gene superfamily, five modes of gene duplication events were identified in pear (*Pyrus* spp*.*) using DupGen_finder software. In total, 1,723 gene pairs were found among the *P450* family genes. The number of DSD-derived gene pairs was highest, followed by WGD- and TD-pairs. Although a large proportion of DSD-pairs were detected in the *P450* gene family, the mechanisms underlying this type of gene duplication remains largely unknown. In total, 117, 88 and 123 gene pairs were inferred to be derived from WGD events in Chinese white pear, European pear and the wild pear, respectively. There were 60, 62 and 73 TD-derived gene pairs in Chinese white pear, European pear and the wild pear, respectively. There were 39 and 37 PD-pairs in Chinese white pear and European pear, but 74 in the wild pear. The numbers of TRD-derived gene pairs in Chinese white pear, European pear and the wild pear were 42, 31 and 30, respectively. The above results demonstrated that the expansion of the *P450* gene family in pear genomes mainly resulted from local single-gene duplication and WGD events. Moreover, the number of *P450* gene pairs that originated from WGD, TD, PD and DSD in cultivated pear were lower than in the wild pear (Fig. 4, Supplementary Table 7a, b).

The *P450* gene pairs were detected between syntenic or homologous chromosomal regions descended from recent WGDs, which occurred before pear and apple split. For example, high percentages of syntenic gene pairs were found between Chr8 and Chr15 in the three pear species, followed by Chr13 and Chr16, Chr6 and Chr14, Chr3 and Chr11, and Chr1 and Chr7 (Supplementary Fig. 2, Supplementary Table 8). The high percentage of WGD-derived gene pairs that have been retained suggests that *P450* family genes may have robust resistance against gene loss during diploidization. The number of syntenic pairs of *P450* genes between European pear and wild pear (335) is highest, contrasting to that of syntenic pairs of *P450* genes between Chinese white pear and European pear (88), as well as between Chinese white pear and wild pear (102) (Fig. 1b, Supplementary Table 8). This may suggest more stronger genome rearrangement and divergence between Chinese white pear and European pear.

### Evolutionary analysis of the *P450* gene family in pear

**Fig. 5:**
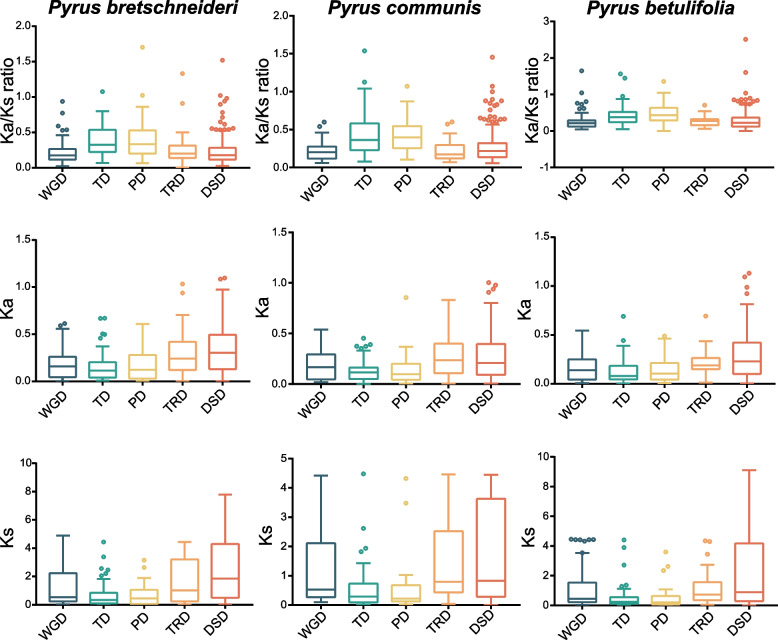
Comparisons of Ka, Ks and Ka/Ks values among different types of duplicated *P450* genes. The *x*-axis shows the five different duplication categories. The *y*-axis shows the Ka, Ks or Ka/Ks ratio. Whole genome duplication (WGD), tandem duplication (TD), proximal duplication (PD), transposed duplication (TRD), dispersed duplication (DSD)

The synonymous (Ks) substitution rate is generally used to estimate the dates of evolutionary events [46]. Approximately 140 million years ago (Mya), a whole-genome triplication event (denoted as γ event) occurred in the ancestor of core eudicots, corresponding to a Ks of 1.5–1.8 [47]. A more recent WGD has been detected in the ancestral lineage of apple and pear, and it has been dated to 30–45 Mya [39]. The Ks values of *P450* gene pairs descended from the WGD events ranged from 0.006 to 4.9 in Chinese white pear, 0.1 to 4.4 in European pear, and 0.002 to 4.5 in the wild pear. There were 28 gene pairs involved in the WGD in Chinese white pear, 26 in European pear and 39 in the wild pear, all having Ks values between 0.15 and 0.3, which suggests that they may have originated from the recent WGD event (30–45 Mya) [39]. In all three pear species, the Ks values of TD- and PD-derived gene pairs were less than those of WGD-derived gene pairs, indicating that they were generated more recently through frequent single-gene duplication events. TRD-derived pairs possessed higher Ks values than WGD-derived pairs, suggesting more ancient origins of the transposed gene duplications (Fig. 5, Supplementary Table 9a—c).

In addition, the Ka/Ks values of homologous gene pairs among the *P450* family genes in the three pear species were calculated. In general, the ratio of Ka (nonsynonymous) to Ks indicated the evolutionary driving forces imposed on a gene. When Ka/Ks > 1, Darwinian selection occurred. When Ka/Ks = 1, neutral selection occurred, and when Ka/Ks < 1, purifying selection occurred [48]. The majority of *P450* gene pairs had Ka/Ks values of less than 1, indicating that these genes have undergone strong purifying selection during evolution to eliminate the deleterious mutations. However, we also found 6, 6 and 10 gene pairs with Ka/Ks values higher than 1 in Chinese white pear, European pear and the wild pear, respectively, suggesting the roles of positive selection in promoting the accumulation of new and favorable mutations. In addition, the Ka/Ks values for TD- and PD-derived gene pairs were higher than for other types of gene pairs, implying that they have evolved at an accelerated rate (Fig. 5, Supplementary Table 9a—c).

### Expression patterns analysis of *P450* genes during pear fruit development

**Fig. 6:**
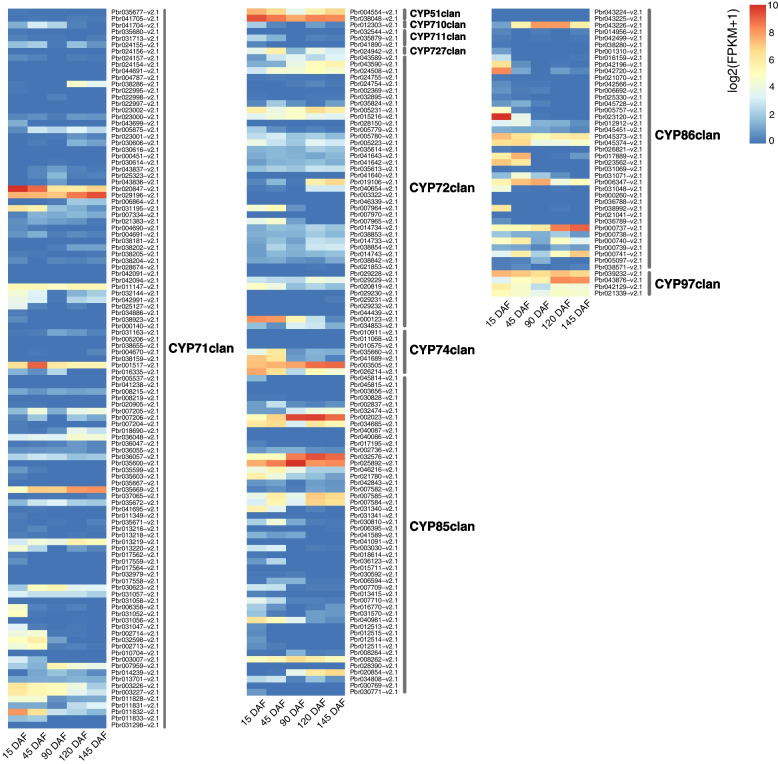
Expression profiles of *P450* genes in Chinese white pear fruit at different developmental stages. RNA-seq data was used to measure the expression levels of *P450* genes. Only genes expressed at least one developmental stage were showed on heatmap. Expression values are indicated by the color scale, with red, yellow and blue indicating high expression, medium and low expression, respectively. DAF, days after flowering

The expression patterns of *P450* genes were investigated using transcriptome data generated from Chinese white pear fruit (‘Dangshansuli’) at five developmental stages (15, 45, 90, 120 and 145 DAF) [46]. The FPKM value was calculated for each *P450* gene to represent the expression level (Supplementary Table 10). We also obtained gene expression data of European pear fruit (‘Bartlett’) at four developmental stages from PearMODB [49] to compare expression patterns of *P450* genes between Chinese white pear and European pear. *P450* genes exhibited higher expression in European pear than in Chinese white pear. The expression of CYP710 clan genes was detected in Chinese white pear, while not in European pear. Expression patterns of P450 genes from the same clan in Chinese white pear and European pear have diverged (Fig. 6 and Supplementary Fig. 8). In total, 79 of 338 *P450* genes of Chinese white pear and 69 of 299 P*450* genes of European pear had no expression at any stage of fruit development, and 144 and 187 genes showed expression at all stages in Chinese white pear and European pear respectively (Supplementary Table 10). Here, we used Chinese white pear P450 genes as representative to clarify the results from expression patterns analysis. Five *P450* genes (*Pbr029196-v2.1*, *Pbr020847-v2.1*, *Pbr025892-v2.1*, *Pbr038048-v2.1* and *Pbr003505-v2.1*) were highly expressed (FPKM > 50) at all stages of fruit development. In particular, the expression levels of 14 genes decreased gradually as the fruit matured, whereas those of 11 genes increased gradually (Fig. 6, Supplementary Table 10). Several families (CYP73, CYP81 and CYP98) in the CYP71 clan showed high expression levels at all stages of fruit development. The CYP51 and CYP97 clans displayed similar expression patterns.

Several models have been proposed to clarify the retention and evolution of duplicated genes, including gene dosage balance, subfunctionalization (SF), neofunctionalization (NF), expression specialization and pseudogenization [47]. Based on the expression profiles, we investigated expression divergence between duplicated genes and explored mechanisms underlying duplicated gene retention. Interestingly, a number of gene pairs showed complementary expression patterns at different fruit developmental stages, which may indicate SF (Supplementary Table 11). For example, the WGD-derived gene pair *Pbr029196-v2.1*–*Pbr020847-v2.1*, in which one gene copy presented high expression levels at several stages, whereas the other showed high expression levels at the remaining stages. Some gene pairs showed parallel expression patterns, indicating that a gene dosage balance was imposed on the evolution of the duplicated pairs to preserve the total expression dosage of ancestral gene. For instance, the TD-derived pairs *Pbr003226-v2.1*–*Pbr003227-v2.1* and *Pbr000740-v2.1*–*Pbr000741-v2.1* presented similar expression patterns at different stages. In addition, expression specialization and nonfunctionalization were found for some gene pairs, such as *Pbr031298-v2.1*–*Pbr003226-v2.1* (TRD-derived pair), *Pbr000260-v2.1-Pbr000737-v2.1* (WGD-derived pair) and *Pbr043225-v2.1*–*Pbr043226-v2.1* (TD-derived pair), in which one gene copy was highly expressed in almost all stages, whereas the other showed low or no expression.

### Identification of candidate *P450* genes involved in flavonoid biosynthesis

**Fig. 7:**
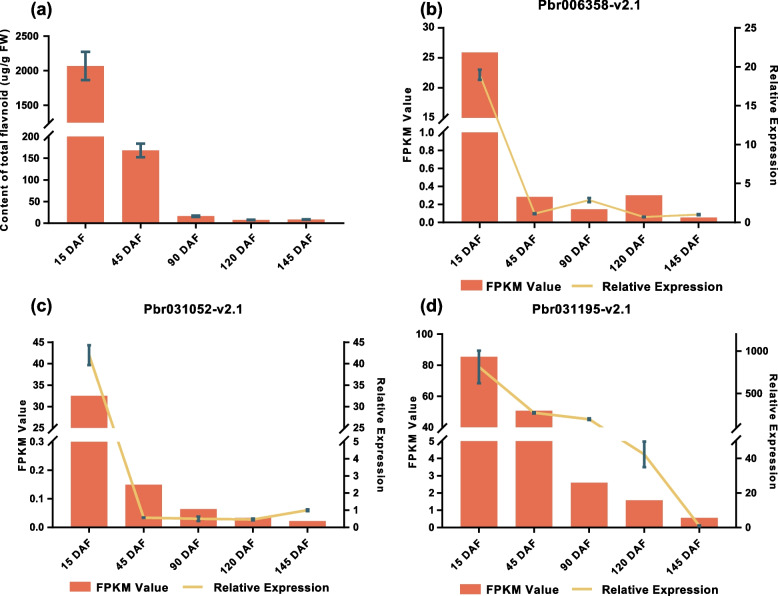
The flavonoid contents and relative expression levels of several *P450* genes at different developmental stages of Chinese white pear fruit. The *x*-axis shows five developmental periods of pear fruits (15, 45, 90, 120 and 145 DAF). Tubulin protein was used as the reference gene to determine expression levels in each period. The *y*-axis represents the flavonoid content, the FPKM (Fragments Per Kilobase of exon model per Million mapped fragments) values of RNA-seq and the relative expression levels calculated using the 2^−△△Ct^ method. The error bars indicate the means ± SDs (*n* = 3). **a** Contents of total flavonoid, **b** *Pbr006358-v2.1*, **c** *Pbr031052-v2.1*, **d** *Pbr031195-v2.1*

We first measured the total flavonoid content in pear fruit (‘Dangshansuli’) at different stages using spectrophotometry, and then, UPLC was used to detect the three major flavonoid components (epicatechin, rutin and myricetin) and their contents (Supplementary Table 12). On the basis of the association analysis between transcriptome expression profile and the total flavonoid content, 23 *P450* genes were screened after removing genes with low expression levels (FPKM < 10). Moreover, the correlated analysis based on Pearson correlation coefficient between the sum of the contents of the three major flavonoid components and their expression profiles was performed, and 19 out of above 23 genes were again identified (two-sided *p*-value (*P*) < 0.05, Supplementary Table 13). Among 23 candidate *P450* genes, 3 genes, belonging to the CYP71 clan, were annotated as flavonoid 3-hydroxylase (*Pbr006358-v2.1* and *Pbr031052-v2.1*) and F3′H (*Pbr031195-v2.1*). F3′H catalyzes dihydrokaempferol or kaempferol to produce dihydroquercetin or quercetin, respectively, and these represent important reactions in the synthetic pathway of myricetin, rutin and epicatechin. Although the gene *Pbr031195-v2.1* was not included in the above 19 gene sets, the correlation coefficient between the expression profile and the sum of the contents of the three major flavonoid components was relatively high (0.87, *P* = 0.054). Moreover, the expression level of *Pbr031195-v2.1* was obviously higher than those of *Pbr006358-v2.1* and *Pbr031052-v2.1*. The expression patterns of the three candidate genes were further verified by qRT-PCR analyses. There was a high consistency between the gene expression trends and the RNA-seq data (Fig. 7). Additionally, we calculated the Pearson’s correlation coefficient between the relative expression of *Pbr031195-v2.1* and total flavonoid component contents based on the UPLC (*r* = 0.96, two-sided *p*-value (*P*) < 0.05). Therefore, *Pbr031195-v2.1* was selected as the target gene to perform subsequent functional research.

### Overexpression and VIGS-mediated gene silencing of a candidate *P450* gene involved in flavonoid biosynthesis in pear fruit

**Fig. 8:**
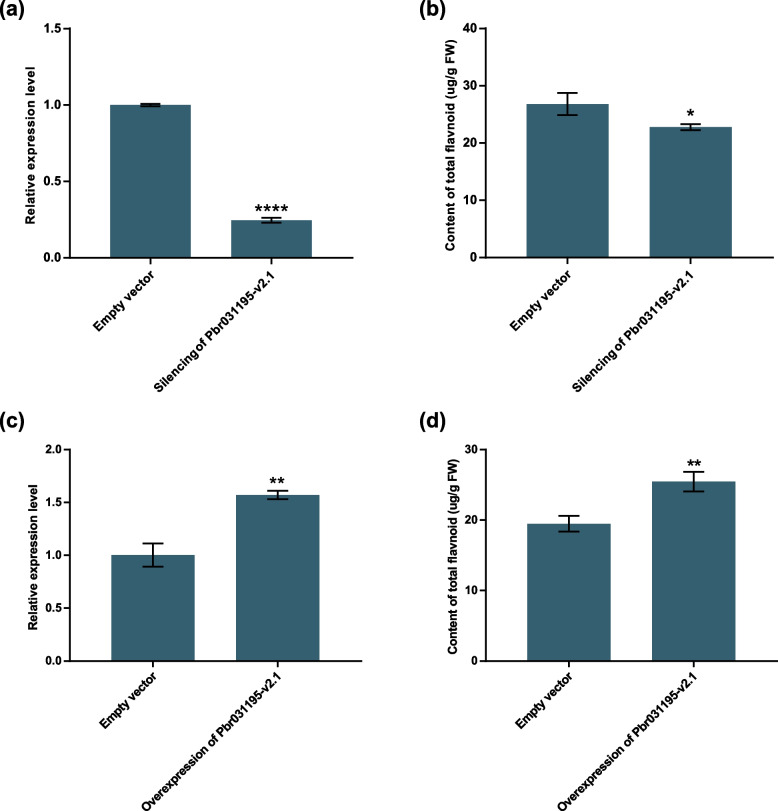
Influence of the transient expression of transformed *Pbr031195-v2.1* on the flavonoid content. **a** The relative expression level of *Pbr031195-v2.1* after transient silencing. **b** Impact of the transient silencing of *Pbr031195-v2.1* expression on the flavonoid content. **c** The relative expression level of *Pbr031195-v2.1* after transient overexpression. **d** Impact of the transient overexpression of *Pbr031195-v2.1* expression on the flavonoid content. Data represent the means ± SDs of three biological replicates. The expression level of *Pbr031195-v2.1* in control fruit was set as 1.0. * Indicates *P*-value < 0.05, ** indicates *P*-value < 0.01 and ****represents *P*-value < 0.0001

To validate the function of *Pbr031195-v2.1* in flavonoid synthesis, we carried out VIGS-mediated gene silencing and the transient overexpression of *Pbr031195-v2.1* in pear fruit. UPLC was used to measure the total flavonoid contents in the transiently transformed samples. The total flavonoid contents of *Pbr031195-v2.1* silenced samples were significantly less than those of samples injected with empty vectors (*P* < 0.05) (Fig. 8b, Supplementary Table 14). In parallel, the relative expression levels of *Pbr031195-v2.1* decreased in the gene-silenced samples (*P* < 0.01) (Fig. 8a). Moreover, in comparison with the samples injected with empty vector, the flavonoid contents significantly increased in the samples overexpressing *Pbr031195-v2.1* (*P* < 0.01) (Fig. 8d, Supplementary Table 14). Additionally, the relative expression levels of *Pbr031195-v2.1* also significantly increased in the gene-overexpression samples (*P* < 0.01) (Fig. 8c). Thus, *Pbr031195-v2.1* expression and flavonoid biosynthesis in pear fruit was positively correlated, indicating that the gene was highly likely to be involved in flavonoid biosynthesis in pear fruit. The molecular functions and regulatory mechanisms of *Pbr031195-v2.1* will be further resolved in a future study.

## Discussion

The *P450* gene superfamily is the largest family of enzyme proteins and they have diverse functions in plants. Its members play catalytic roles in various biochemical reactions, including the biosynthesis of flavonoids, abscisic acid, phenylpropanol and brassinolide [9, 12, 19]. Members of *P450* gene superfamily have been identified and analyzed in a variety of plants [8, 13, 19, 42], and the number and categories of *P450* family genes varies greatly [5, 12]. However, studies on the origin, evolution and functional diversification of the *P450* genes in pear and Rosaceae species remain lacking. Here, we identified a repertoire of *P450* genes in cultivated and wild pear genomes for the first time. The number of *P450* genes (299–419) in pear is higher than in *A. thaliana* (272), *C. papaya* (142), *S. lycopersicum* (233), *M. notabilis* (174) and Tartary buckwheat (285) [7, 8, 11, 13, 19]. Wild pear possessed the highest number of *P450* genes, which may play important roles in robust adaptation of wild pear to harsh conditions. In accordance with the classification rules of *P450* genes in other species [2], the pear *P450* family genes were divided into ten clans belonging to two types, A-type and non-A-type. The number of A-type genes is higher than non-A-type genes in plants [7, 11, 16, 42, 50, 51]. Similarly, more *P450* A-type genes were found in European pear (155 vs 144) and the wild pear (225 vs 194), but the opposite was true in Chinese white pear (152 vs 186).

Most of the *P450* genes in the same family or subfamily have similar functions, whereas the genes from different families show distinct functional roles [5, 10, 12, 45]. A number of *P450* families may be involved in the biosynthesis of secondary metabolites, such as terpenoids, flavonoids, steroids, alkaloids and phenylpropanoids. For example, the CYP51, CYP82 and CYP706 families are involved in the biosynthesis of antimicrobial triterpenes, homoterpene volatiles and sesquiterpenes [23, 44, 52]. The roles of *P450* families in the biosynthesis of hormones, such as auxin, gibberellin and brassinosteroid, have been well documented. For instance, the CYP79 and CYP83 families participate in auxin biosynthesis [53, 54], whereas the CYP85, CYP90, CYP72, CYP724 and CYP734 families are responsible for the deactivation or catabolism of brassinosteroid hormones [45, 55–58].

The number of *P450* genes in the same clan or family varies greatly among different plant species, which may be related to the specialized evolution of *P450* genes in different lineages. Five single-family clans (CYP51, CYP74, CYP97, CYP710 and CYP746) and the CYP86 family were found in chlorophytes and charophytes, suggesting their ancient origins [5]. The appearance of two multiple-family clan (CYP71 and CYP85) members and another single-family clan (CYP711) accompanied the origin of moss [5]. CYP71 is a large clan that contains a number of families, and it is thought to have originated in land plants and has expanded in a number of extant plant genomes [59]. In addition, the CYP746 family (CYP746 clan) has not been detected in vascular plants but has been found in algae and moss [10]. In this study, the members of the CYP99 and CYP723 clans were only observed in rice (monocot). Some lineage-specific CYP families evolved during the diversification of seed plants. For example, the CYP725 family (clan CYP85) may be specific to *Taxus* species, CYP750 (clan CYP71) is gymnosperm-specific, and CYP719 (clan CYP71) is Ranunculales-specific. Nevertheless, the lineage-specific loss of some CYP families was also found in pear and other plants in this study. For example, the CYP82 family (clan CYP71) was found in pear, Arabidopsis, grape, tomato and sacred lotus but not in rice. The CYP733 (clan CYP85) and CYP736 (clan CYP71) families exist in grape, papaya, morus and tomato but have been lost in pear and Arabidopsis. The CYP705 family (clan CYP71) is present in pear and Arabidopsis but is absent from citrus, grape, tomato, sacred lotus and rice.

The conserved motif and gene structure analyses of pear *P450* genes revealed similar patterns of conserved motifs and intron–exon structures among members of the same clan, supporting phylogenetic classification of *P450* genes. A number of pear *P450* genes contained only one exon (57 of 338, 16.9%) or two exons (128 of 338, 37.9%), which revealed simple gene structures compared with other gene families identified in pear, such as the *bHLH* [60], *RNase T2* gene [61], superoxide dismutase gene [62] and polygalacturonase gene [63] families. It has been suggested that single-exon genes (SEGs) are produced by the retro-transposition of multi-exon genes [64], and their evolutionary rates are usually higher than those of intron-containing genes [65]. In the dinoflagellate *Polarella glacialis*, the SEGs are associated with survival in extreme environments and have enhanced the total expression dosage in stress responses through TD [66].

The expansions of gene families are the drivers of genome and gene duplication events, including WGD, TD, PD, TRD and DSD events [19]. Gene duplication provides genetic material for evolving new functions or ancestral functional partitioning between duplicated gene pairs, which promotes plant adaptation to the changing environment [67, 68]. The contributions of different gene duplication events to the expansions of gene families vary. In this study, WGDs and small-scale gene duplications (TDs and PDs) were found to have made large contributions to the expansion of *P450* gene superfamily in pear. The high number of *P450* genes in pear compared with the number in Arabidopsis and other plants can be attributed to the recent lineage-specific WGD event occurring in the ancestor of Maloideae (now defined as Amygdaloideae) [39]. In pear genome, a large amount of WGD-derived *P450* gene pairs were retained during long-term evolution, and they escaped from the genome reshuffling and gene loss that occurred shortly after the WGD. The Ka/Ks values of most paralogous gene pairs in the pear *P450* gene family are less than 1, indicating that they experienced strong purifying selection. Several gene pairs with Ka/Ks > 1 were also found, indicating that positive selection plays a role in fixing favorable mutations and in the functional diversification of evolution. These positively selected genes were related to flavonoid 3’-monooxygenase, flavonoid 3’,5’-hydroxylase and some oxidoreductases, such as steroid 3-oxidase [39].

*P450* family genes play essential roles in plant growth and development, and they catalyze various reactions during the biosynthesis of a variety of secondary metabolites, such as flavonoids, steroids, terpenoids, phenylpropanoid and alkaloids [12]. Flavonoids, the largest secondary metabolite family in plants, have more than 10,000 structures and different antioxidant effects, which play important roles in the anti-stress processes of plants [69]. Additionally, they contribute to the nutrient supply, human health and pharmacology [70]. At present, many genes or transcription factors have been reported to be involved in the biosynthesis of flavonoids in the plant kingdom, such as chalcone synthase, chalcone isomerase, flavanone 3-hydroxylase, flavone synthase I, *F3′H*, R2R3-MYB, bHLH and NF-YB [19, 33, 71–75]. The scaffold structures of flavonoids may have been preliminarily formed by enzymes from primary metabolic pathways and were then modified by enzymes belonging to superfamilies, such as the cytochrome *P450* family [33]. In this study, flavonoid contents and gene expression patterns at different developmental stages of pear fruit were investigated. In total, 144 *P450* genes were expressed during all fruit developmental stages of Chinese white pear. Three candidate *P450* genes involved in flavonoid biosynthesis were further identified through an association analysis between flavonoid content and transcriptome expression profiles. These genes were all identified as members of the CYP71 clan. A gene member (*Pbr031195-v2.1*) of the CYP75 family was found to be closely associated with flavonoid content changes as determined by qRT-PCR, overexpression and gene silencing analyses. However, the transcription factors that regulate this key gene remain unexplored. Further studies should focus on the molecular regulatory mechanisms of candidate genes involved in flavonoid metabolism.

## Conclusion

In summary, 338, 299 and 419 *P450* genes were comprehensively identified in the genomes of Chinese white pear, European pear and the wild pear, respectively. On the basis of a phylogenetic analysis, *P450* genes in pear were classified into ten clans (CYP51, CYP71, CYP72, CYP74, CYP85, CYP86, CYP97, CYP710, CYP711 and CYP727) and 48 families. WGDs and small-scale duplications largely contributed to the *P450* gene family expansion in pear. The *P450* genes underwent strong purifying selection. The candidate genes (*Pbr031195-v2.1*, *Pbr006358-v2.1* and *Pbr031052-v2.1*) related to flavonoid metabolism were screened using a correlated analysis between the dynamic transcriptome and flavonoid contents, and the function of one gene was validated using a combination of qRT-PCR and transient transformation. These results are valuable as a basis for further studies on the molecular functions of *P450* genes in pear.

## Methods

### Plants materials

In this experiment, the pear trees (*Pyrus bretschneideri* cv. ‘Dangshansuli’) were cultivated in a pear germplasm orchard in Nanjing, Jiangsu Province, China. The fruit samples at different developmental stages [15, 45, 90, 120, and 145 days after flowering (DAF)] were picked from different trees. The samples were ground in liquid nitrogen and stored at − 80 °C until use.

### Sequence identification of *P450* genes

The genome assembly and annotation files of European pear (*P. communis*) and the wild pear ‘Shanxi Duli’ (*P. betulifolia*) were attained from the GDR database (https://www.rosaceae.org). An improved genome of Chinese white pear (*P. bretschneideri*) was used in this research (unpublished data). The *P450* protein sequences of Arabidopsis were downloaded from the Arabidopsis Cytochromes *P450* database (http://www.P450.kvl.dk/) [76]. Additionally, the *P450* protein sequences of tomato (*Solanum lycopersicum*) and grape (*V. vinifera* L.) were retrieved from the Cytochrome *P450* Homepage (http://drnelson.uthsc.edu/CytochromeP450.html) [2].

In this study, two approaches were applied to identify the cytochrome *P450* genes. First, the seed alignment file for the characteristic domain (Pfam ID: PF00067) of the *P450* gene family was downloaded from the Pfam website (https://pfam.xfam.org/) [77], and hmmsearch, implemented in HMMER3 software, was used to search for candidate *P450* sequences against the pear whole-genome protein database [78]. Second, the protein sequences of *P450* genes in Arabidopsis, tomato and grape served as query sequence to search for candidate *P450* genes using the BLASTP algorithm implemented in DIAMOND software with E-values < 1e^−10^ [79]. The sequences contained in both candidate *P450* gene sets resulting from the two methods were considered as high-confidence *P450* genes. Fragmented sequences with amino acid length less than 100, or those without the *P450* characteristic domain, were removed based on the reconfirmation on the Pfam website. Basic information on *P450* sequences, including amino acid length, molecular weight (MW) and isoelectric point (pI) were analyzed using the ProtParam tool (http://web.expasy.org/compute_pi/).

### Multiple sequence alignment and phylogenetic analysis

We downloaded *P450* representative protein sequences of each plant subfamilies from the Cytochrome *P450* Homepage [2]. MAFFT was used to execute multiple sequence alignments of above protein sequences and pear *P450* sequences [80]. Then, IQ-TREE was used to build a maximum-likelihood phylogenetic tree [81]. The bootstrap values were set to 1,000. The pear *P450* genes were classified into different clans, families and subfamilies on the basis of classification criterion from the Cytochrome *P450* Homepage and the phylogenetic tree [2].

### Gene structure and conserved motif analysis of *P450* family members

TBtools was used to present the exon–intron structures of the *P450* genes [82]. The MEME (http://meme-suite.org/tools/meme) tool was utilized to identify the conserved motifs of *P450* genes [83]. In total, 10 conserved motifs were analyzed, and the default parameters were applied.

### Collinearity and evolutionary analyses of *P450* family genes

The DupGen_finder pipeline was used to identify diverse modes of gene duplication in the pear genome [84]. MCScanX software was implemented to identify the collinear relationships within the pear genome and collinear gene pairs among *P450* superfamily members [85]. We used TB-tools software to visualize the above results [82]. The calculate_Ka_Ks_pipeline was utilized to calculate Ka, Ks and the Ka/Ks ratio [47]. Briefly, the computing_Ka_Ks_pipe.pl script was performed to complete sequence alignments with MAFFT for each gene pair. Then, we computed Ka and Ks values with the GMYN model using the KaKs_Calculator 2.0 software and the alignment file converted to the AXT format as the input file [86].

### RNA isolation, library construction and sequencing

Total RNA was extracted from pear fruits of different developmental stages with three replicates using the RNAprep Pure Plant Kit (Polysaccharides & Polyphenolics-rich) (Tiangen, Beijing, China). Fifteen libraries were constructed using NEBNext UltraTM RNA Library Prep Kit for Illumina (NEB, MA, USA) following manufacturer’s recommendations. Sequencing was performed on an Illumina platform.

### Transcriptome and expression analyses of *P450* genes

RNA-seq was conducted for ‘Dangshansuli’ pulp collected from different developmental stages (15, 45, 90, 120 and 145 DAF) with three replicates [87]. Adapter, ploy-N and low-quality reads were removed from the raw reads to produce clean reads. We used Hisat2 to construct an index of the reference genome and align paired-end clean reads with the reference genome [88]. The read numbers that were mapped to each gene were calculated using FeatureCounts [89]. Then, the Fragments Per Kilobase of exon model per Million mapped fragments (FPKM) value for each gene was computed to represent the gene expression level. We downloaded gene expression data of European pear (‘Bartlett’) fruit at different developmental stages from PearMODB [49]. Heatmap was plotted using R package ‘pheatmap’.

### Measurement and quantification of the flavonoid content and components in pear

The flavonoid contents of pear fruit (cv. ‘Dangshansuli’) at different developmental stages were measured using two methods. Before the measurement, we first extracted the sample solution. Accurately weigh 1.0 g of the sample powder frozen at -80 ºC, add 3 mL of extraction solvent (80% methanol solution), vortex and mix for 30 s, put it in an ultrasonic cleaner adjusted to 100 Hz, and sonicate for 30 min. Shake once every 5 min, 30 s each time, take it out and let it stand for 10 min, take the supernatant, centrifuge at 12,000 r/min for 10 min, and filter it with a 0.22 μm filter membrane to obtain the sample solution. The total flavonoid content was determined by spectrophotometry. Rutin was used as a standard to measure the absorbance of pear pulp sample solutions at 510 nm. Take 0.1 mL sample solution, and add 0.1 mL 5% NaNO_2_ solution. Mix the solution thoroughly and let stand at room temperature for 6 min. Next, add 0.1 mL 10% Al(NO_3_)_3_ solution, shake well and continue to stand for 6 min. Then, introduce 0.3 mL 1 mol/L NaOH solution and 0.4 mL 80% methanol solution, ensuring thorough mixing. After standing for 15 min, measure the absorbance value at 510 nm. Simultaneously, use 0.1 mL 80% methanol solution as blank control. Prepare rutin standard solutions with varying concentration gradients and follow the above steps to determine the absorbance values. Construct a standard curve based on the obtained data. The total flavonoid content was expressed as the amount of rutin per gram of the sample. The flavonoid components and contents were measured using Ultra-Performance Liquid Chromatography (UPLC). The UltiMate3000 system and an ACQUITY UPLC HSS T3 C18 column (2.1 mm × 100 mm, 1.8 μm) were used with a mobile phase of 0.1% acetic acid and acetonitrile. The following parameters were set: column temperature, 30 °C; wavelength, 288 nm; flow rate, 0.35 mL·min^−1^; and sample quantity, 2 μL. We obtained the flavonoid content by substituting the peak area of the sample into the standard curve equation. Each sample with three replicates were used in above two methods.

### Quantitative real-time PCR (qRT-PCR) analysis

Total RNA from five developmental stages of ‘Dangshansuli’ pulp were extracted using a Plant Total RNA Isolation Kit Plus (Fuji, China). TransScript One-Step gDNA Removal and cDNA Synthesis SuperMix (TransGen, China) were used to attain cDNA. The LightCycler 480 SYBRGREEN I Master (Roche, USA) was used to perform qRT-PCR. The PCR reaction system consisted of 1 μL of sense and anti-sense primer (10 μM), 5 μL of 2 × SYBR Premix ExTaq TM, 1 μL of cDNA template and 3 μL of sterilized water. The qRT-PCR procedure was as follows: 10 min at 95 °C, followed by 45 cycles of 95 °C for 3 s, 60 °C for 10 s and 72 °C for 30 s. The 2^−△△ct^ method was used to measure the relative expression. Primer Premier 6.0 was used to design the primers for all the selected genes (PREMIER Biosoft International, USA) and the “Tublin” gene was used as endogenous control gene (Supplementary Table 15).

### Transient transformation of pear fruit

An approximately 300-bp coding sequence at the C-terminus of *Pbr031195-v2.1* was amplified and then inserted into the pTRV2 vector [63]. We introduced the constructed pTRV2-*Pbr031195-v2.1* vector and pTRV1 independently into *Agrobacterium tumefaciens* strain GV3101, and then, centrifuged the incubated suspensions before resuspended them in the infiltration buffer (10 mM MgCl_2_, 10 mM MES, pH 5.5, and 150 μM acetosyringone). The 1:1 mixtures of *A. tumefaciens* that contained pTRV2-*Pbr031195-v2.1* and pTRV1 were injected independently into pear flesh tissue [68]. The empty pTRV2 vector and pTRV1 were co-injected as control. In addition, we also prepared a p1300-35S-GFP-BS2 vector to overexpress *Pbr031195-v2.1* (35S-*Pbr031195-v2.1*-GFP). After transforming the overexpression vector into *A. tumefaciens* strain GV3101, the incubation temperature was set to 28 °C, and the OD_600_ value was adjusted to 0.6–0.8 [62]. We resuspended the bacterial strain in buffer after centrifugation, and then injected it into the pulp tissue of detached fruit of 90DAF using a sterile syringe. Injected fruit were stored at 25 °C for 5 days, and then the pulp around the injection hole were collected and ground in liquid nitrogen. There were three replicates per vector, with five fruits per replicate.

### Supplementary Information

**Additional file 1: Table 1a.** Gene features of P450 family members in Chinese white pear. **Table 1b.** Gene features of P450 family members in European pear. **Table 1c.** Gene features of P450 family members in the wild pear.

**Additional file 2: Figure 1.** Length distribution of P450 protein in pear (Pyrus spp.).

**Additional file 3: Figure 2.** P450 genes in three pear species were mapped to different chromosomes. The collinearity relationships of gene pairs are represented by red lines. Different chromosomes are represented by different colors. Different colored rectangles represent gene clusters on different chromosomes. (a) Chinese white pear, (b) European pear, (c) The wild pear.

**Additional file 4: Table 2.** The chromosomal distribution of P450 genes in pear (Pyrus spp.).

**Additional file 5: Table 3a.** The P450 gene clusters identified in Chinese white pear. **Table 3b.** The P450 gene clusters identified in European pear. **Table 3c.** The P450 gene clusters identified in the wild pear.

**Additional file 6: Figure 3.** Phylogenetic analysis of P450 genes in Chinese white pear and four Rosaceae species (Malus domestica, Fragaria vesca, Prunus persica and Prunus mume).

**Additional file 7: Table 4a.** Classification of A-type cytochrome P450 genes in Chinese white pear. **Table 4b.** Classification of non-A-type cytochrome P450 genes in Chinese white pear.

**Additional file 8: Figure 4.** Phylogenetic analysis of P450 genes in Chinese white pear, European pear and wild pear.

**Additional file 9: Table 5.** Distribution of P450s in different plant species that had been reported.

**Additional file 10: Table 6a.** Motif sequences of P450s identified by MEME tools in three pears. **Table 6b.** Motif sequences of P450s identified by MEME tools in Chinese white pear. **Table 6c.** Motif sequences of P450s identified by MEME tools in European pear. **Table 6d.** Motif sequences of P450s identified by MEME tools in the wild pear.

**Additional file 11: Figure 5.** Motifs and gene structure of P450 genes in European pear.

**Additional file 12: Figure 6.** Motifs and gene structure of P450 genes in wild pear.

**Additional file 13: Figure 7.** Motifs and gene structure of P450 genes in three pears.

**Additional file 14: Table 7a.** Number of different modes of gene duplication events identified in P450 superfamily in pear.

**Additional file 15: Table 8.** Collinearity relationships among P450 genes within pear genomes.

**Additional file 16: Table 9a.** Ka/Ks values of duplicated P450 gene pairs in Chinese white pear. **Table 9b.** Ka/Ks values of duplicated P450 gene pairs in European pear. **Table 9c.** Ka/Ks values of duplicated P450 gene pairs in the wild pear.

**Additional file 17: Table 10a.** The expression levels (FPKM) of P450 genes in Chinese white pear fruit. **Table 10b.** The expression level (TPM) of P450 genes in European pear fruit.

**Additional file 18: Figure 8.** Expression profiles of P450 genes in European pear fruit at different developmental.

**Additional file 19: Table 11.** The retention mechanisms of P450 duplicated gene pairs inferred from transcriptome profiles in Chinese white pear.

**Additional file 20: Table 12.** Content of total flavonoid at different developmental stages of Chinese white pear.

**Additional file 21: Table 13.** The Pearson correlation coefficient between the FPKM value of the selected genes and total flavonoid content at different developmental stages in Chinese white pear.

**Additional file 22: Table 14.** The flavonoid content of pear fruit after transient transformation of Pbr031195-v2.1.

**Additional file 23: Table 15.** The primers for qRT-PCR of candidate genes in Chinese white pear P450 gene family.

## Data Availability

All data used in this study are included in this article and additional flies. The RNA-seq data used in this study has been deposited on NGDC-GSA (CRA012640) [90, 91], and can be accessed through https://ngdc.cncb.ac.cn/gsa/browse/CRA012640.

## Abbreviations

CYP: Cytochrome *P450* monooxygenase
MYA: Million years ago
HMM: Hidden Markov Model
ML: Maximum likelihood
MEME: Multiple EM for Motif Elicitation
DSD: Dispersed duplication
PD: Proximal duplication
TD: Tandem duplication
TRD: Transposed duplication
WGD: Whole-genome duplication
DAF: Days after flowering
MW: Molecular weight
pI: Isoelectric point
UPLC: Ultra Performance Liquid Chromatography
Chr: Chromosome
FPKM: Fragments Per Kilobase of exon model per Million mapped fragments
GSDS: Gene Structure Display Server
MEME: Multiple Expectation Maximization for Motif Elicitation
qRT-PCR: Quantitative real-time polymerase chain reaction
VIGS: Virus-induced gene silencing
Ks: Synonymous substitutions per site
Ka: Nonsynonymous substitutions per nonsynonymous site
SF: Subfunctionalization
NF: Neofunctionalization
SEGs: Single exon genes
