# Supplementary material for: Genome-wide identification, comparative analysis and functional roles in flavonoid biosynthesis of cytochrome P450 superfamily in pear (Pyrus spp.)
Source: BMC Genom Data. 2023 Oct 3;24:58. doi: 10.1186/s12863-023-01159-w (PMC10548706; doi:10.1186/s12863-023-01159-w)

Supplementary Figure1. Length distribution of P450 protein in pear (Pyrus spp.). We have shown the distribution of amino acid lengths corresponding to the PbCYP genes, with the horizontal axis representing the number of amino acids corresponding to the PbCYP genes and the vertical axis representing the number of genes.

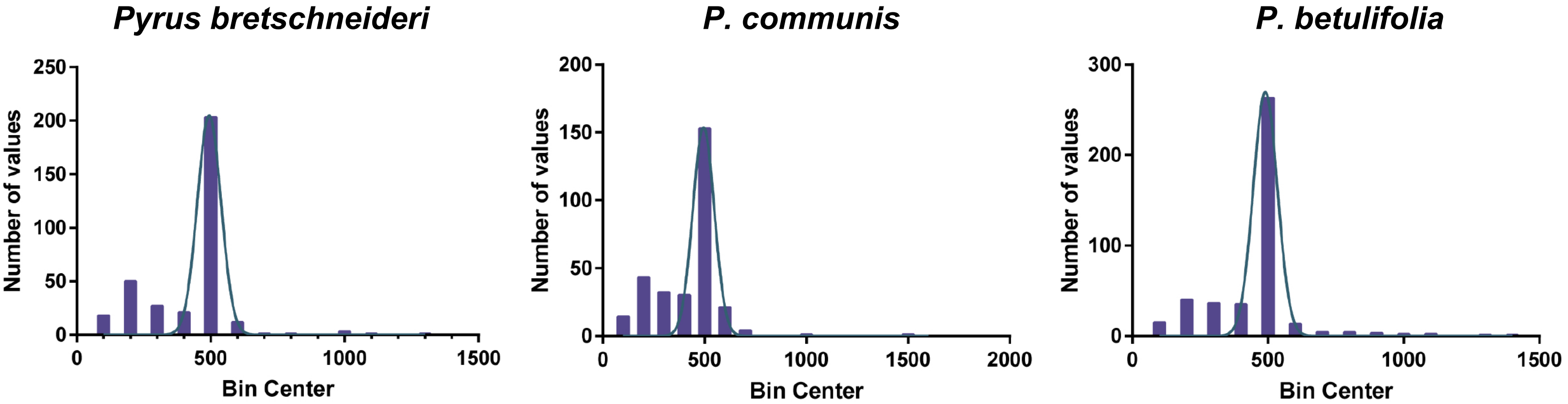

Supplement: Supplementary file 2 — Additional file 2: Figure 1. Length distribution of P450 protein in pear (Pyrus spp.). [file 12863_2023_1159_MOESM2_ESM.pdf]
