# Supplementary material for: Genome-wide identification, comparative analysis and functional roles in flavonoid biosynthesis of cytochrome P450 superfamily in pear (Pyrus spp.)
Source: BMC Genom Data. 2023 Oct 3;24:58. doi: 10.1186/s12863-023-01159-w (PMC10548706; doi:10.1186/s12863-023-01159-w)

Supplementary Figure 2. Gene localization and syntenic relationships of the P450 genes in the three pear genomes.

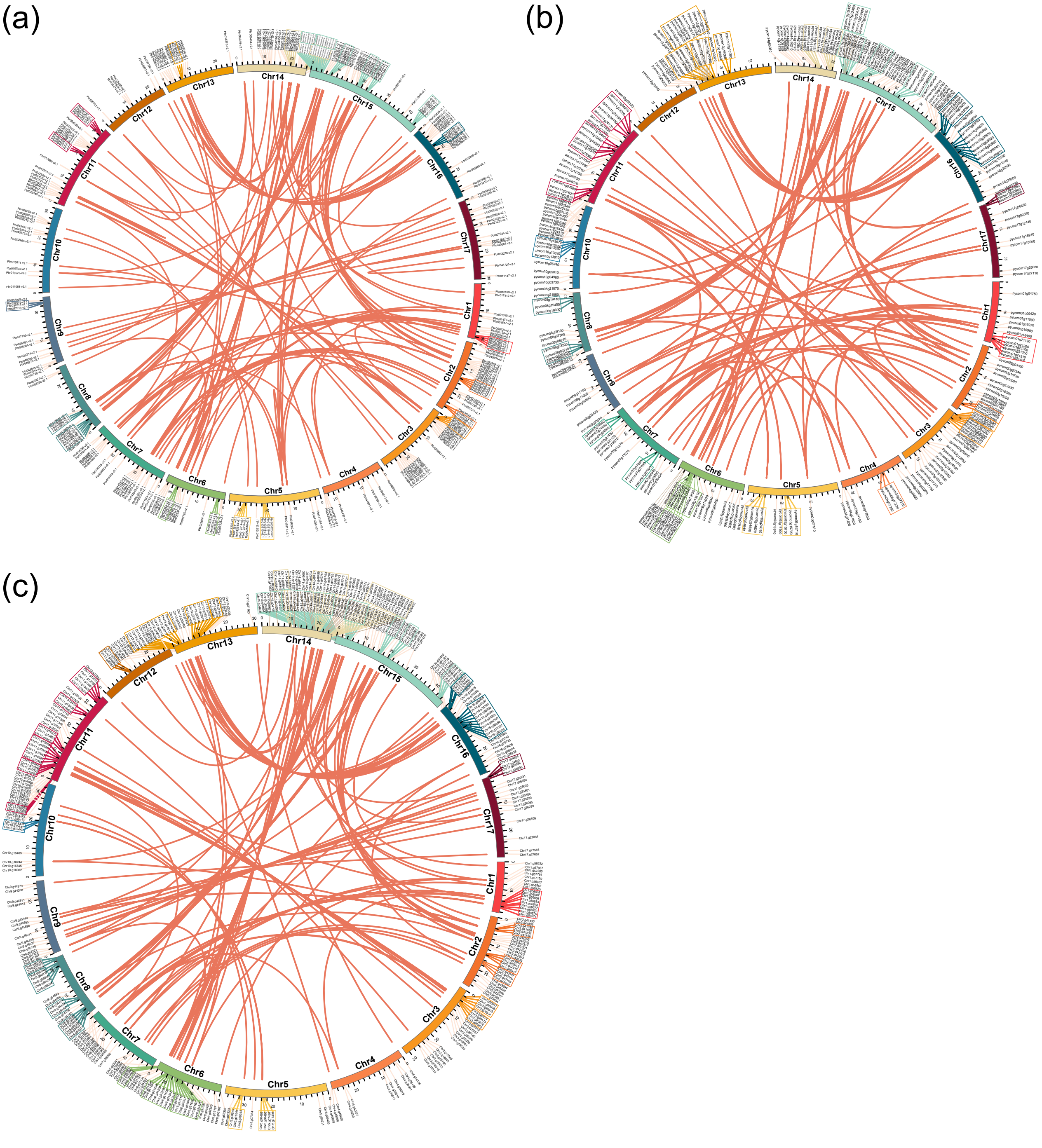

Supplement: Supplementary file 3 — Additional file 3: Figure 2. P450 genes in three pear species were mapped to different chromosomes. The collinearity relationships of gene pairs are represented by red lines. Different chromosomes are represented by different colors. Different colored rectangles represent gene clusters on different chromosomes. (a) Chinese white pear, (b) European pear, (c) The wild pear. [file 12863_2023_1159_MOESM3_ESM.pdf]
