# Supplementary material for: Genome-wide identification, comparative analysis and functional roles in flavonoid biosynthesis of cytochrome P450 superfamily in pear (Pyrus spp.)
Source: BMC Genom Data. 2023 Oct 3;24:58. doi: 10.1186/s12863-023-01159-w (PMC10548706; doi:10.1186/s12863-023-01159-w)

Supplementary Figure 4. Phylogenetic analysis of P450 genes in Chinese white pear, European pear and wild pear.

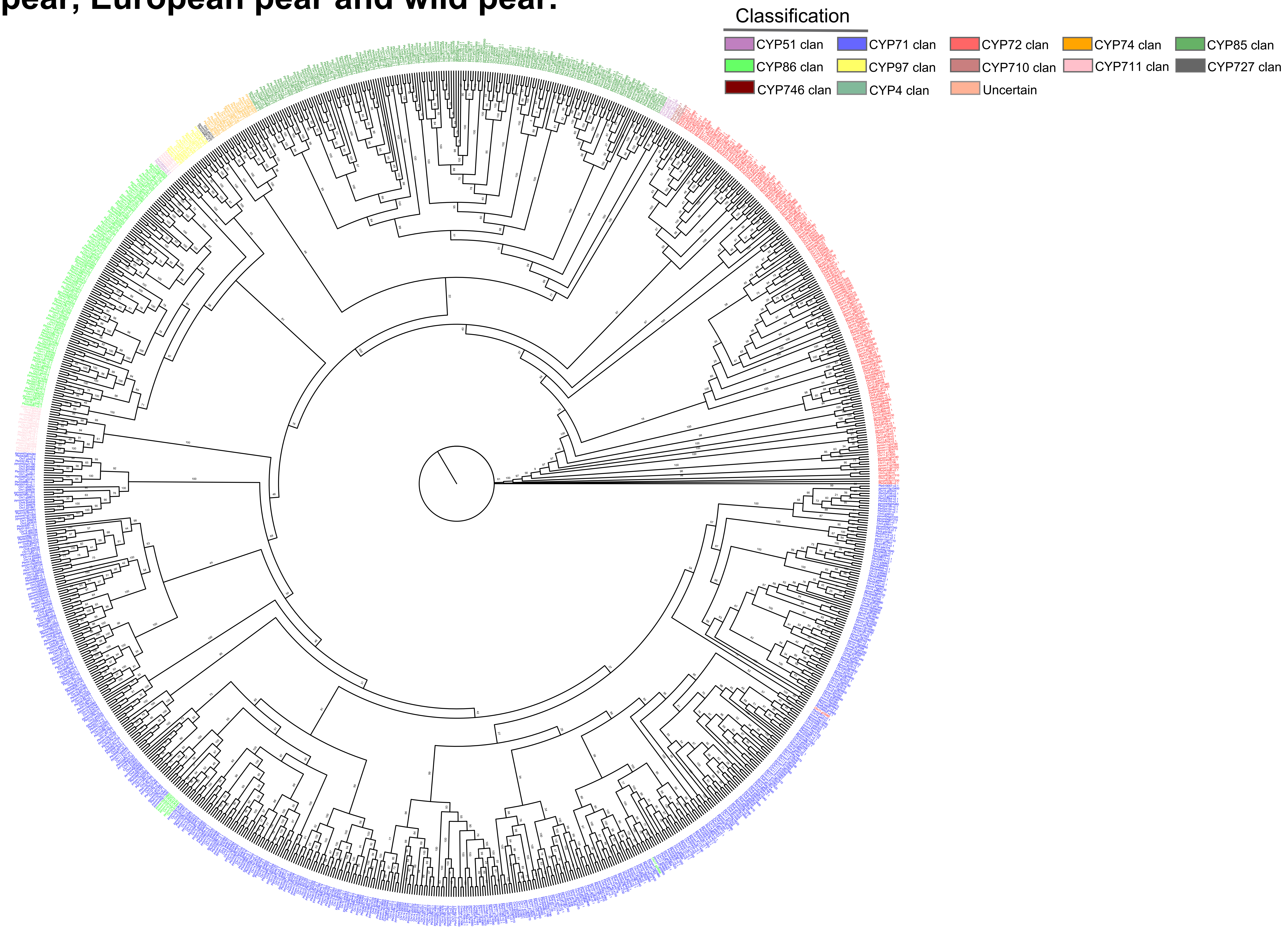

Supplement: Supplementary file 8 — Additional file 8: Figure 4. Phylogenetic analysis of P450 genes in Chinese white pear, European pear and wild pear. [file 12863_2023_1159_MOESM8_ESM.pdf]
