# Supplementary figures and images for: Genome-wide identification, comparative analysis and functional roles in flavonoid biosynthesis of cytochrome P450 superfamily in pear (Pyrus spp.)
Source: BMC Genom Data. 2023 Oct 3;24:58. doi: 10.1186/s12863-023-01159-w (PMC10548706; doi:10.1186/s12863-023-01159-w)

Supplementary Figure 5. Motifs and gene structure of P450 genes in European pear.

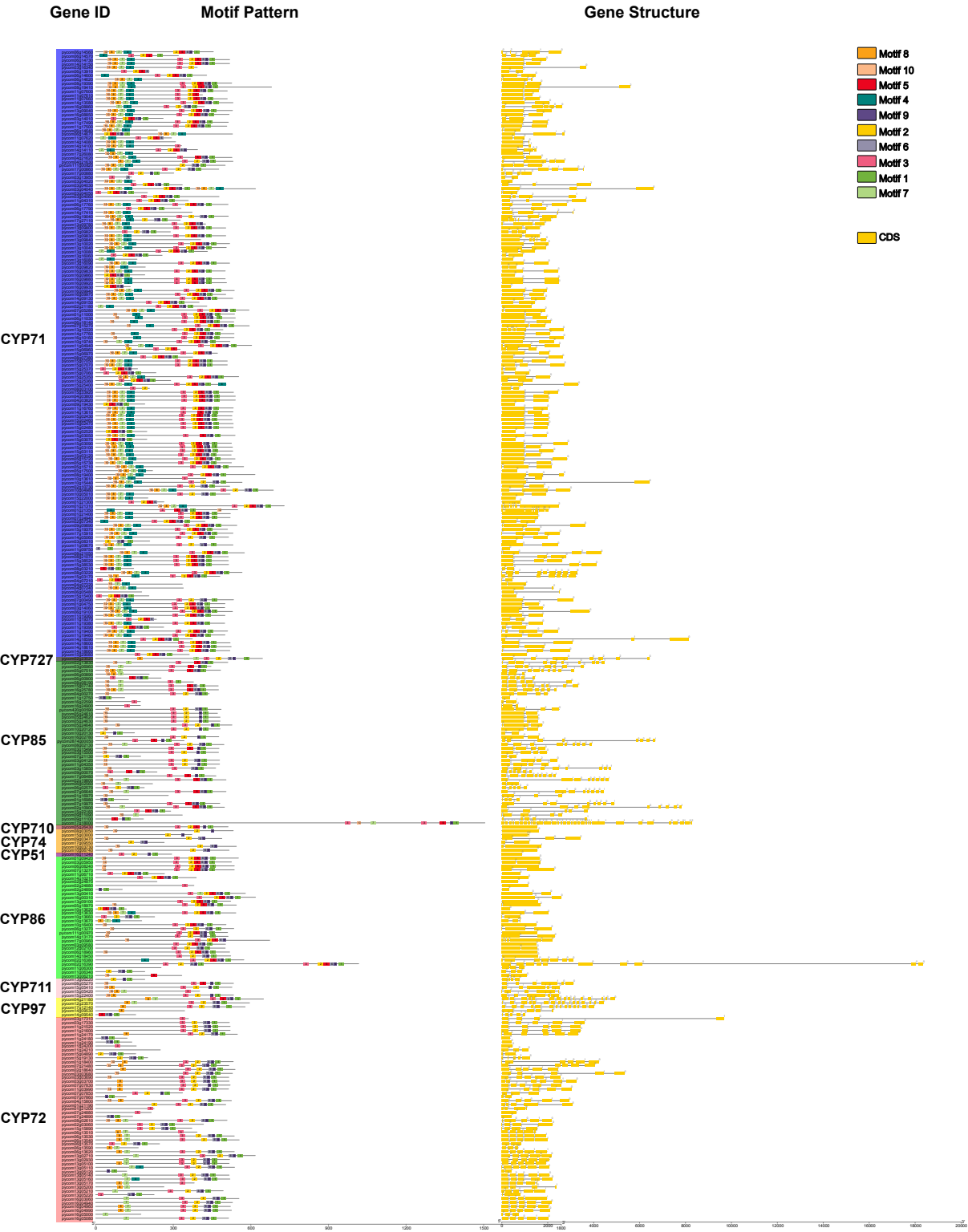

Supplement: Supplementary file 11 — Additional file 11: Figure 5. Motifs and gene structure of P450 genes in European pear. [file 12863_2023_1159_MOESM11_ESM.pdf]

Supplementary Figure 6. Motifs and gene structure of P450 genes in wild pear.

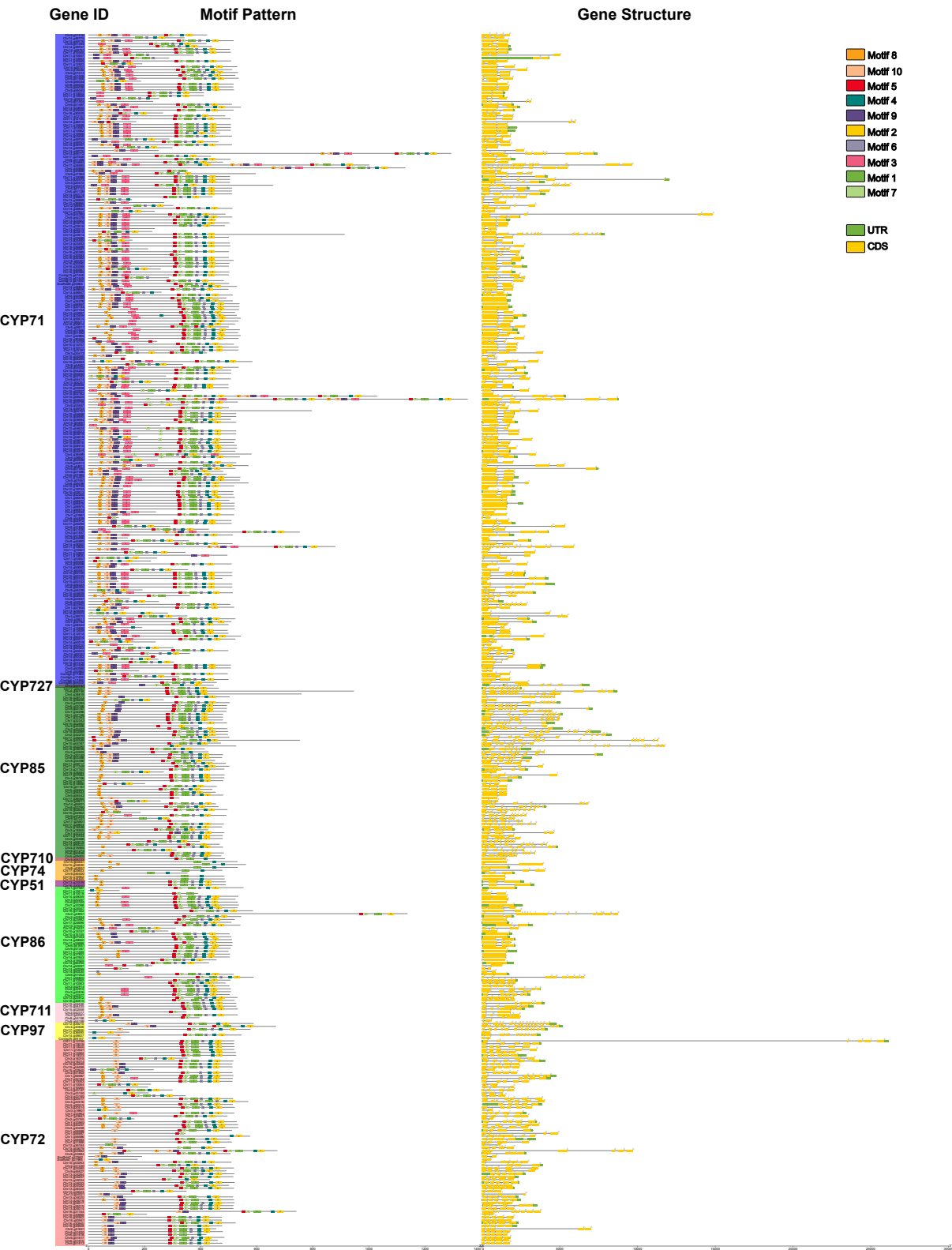

Supplement: Supplementary file 12 — Additional file 12: Figure 6. Motifs and gene structure of P450 genes in wild pear. [file 12863_2023_1159_MOESM12_ESM.pdf]

Supplementary Figure 7. Motifs and gene structure of P450 genes in three pears.

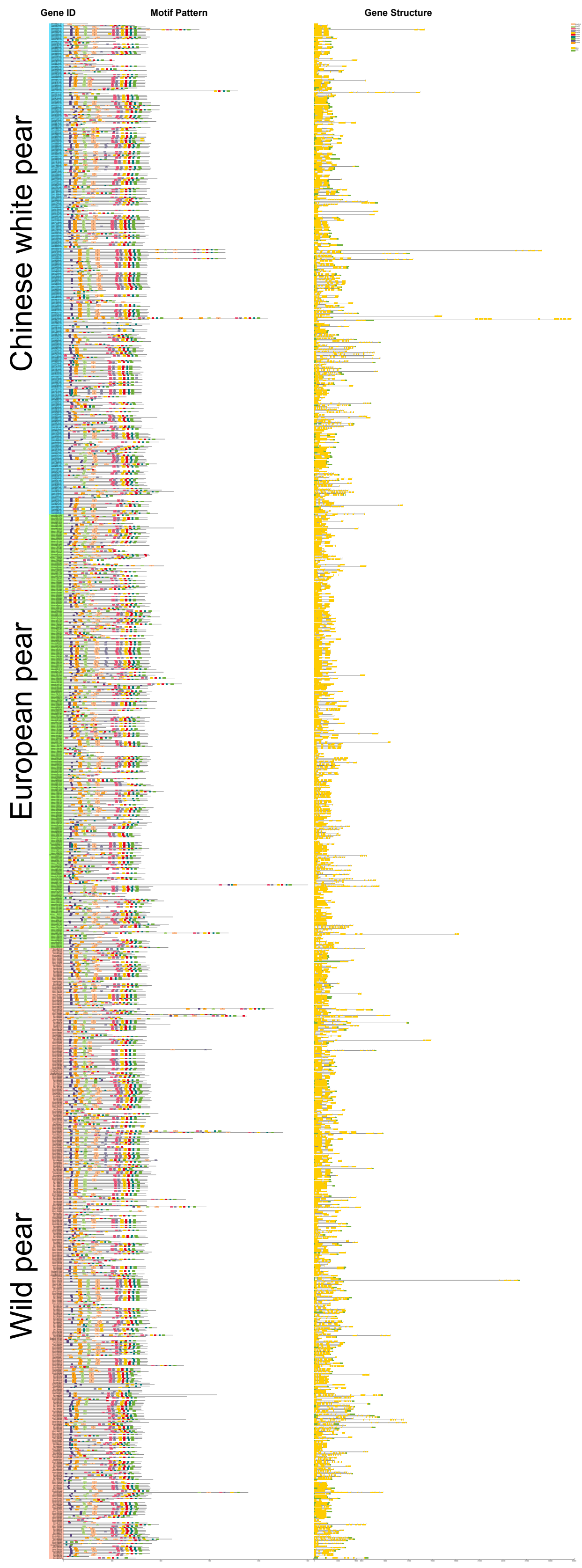

Supplement: Supplementary file 13 — Additional file 13: Figure 7. Motifs and gene structure of P450 genes in three pears. [file 12863_2023_1159_MOESM13_ESM.pdf]
